# Glabridin, a Bioactive Flavonoid from Licorice, Effectively Inhibits Platelet Activation in Humans and Mice

**DOI:** 10.3390/ijms231911372

**Published:** 2022-09-27

**Authors:** Chi-Li Chung, Jui-Hsuan Chen, Wei-Chieh Huang, Joen-Rong Sheu, Chih-Wei Hsia, Thanasekaran Jayakumar, Chih-Hsuan Hsia, Kuan-Rau Chiou, Shaw-Min Hou

**Affiliations:** 1 Division of Pulmonary Medicine, Department of Internal Medicine, Taipei Medical University Hospital, Taipei 110, Taiwan; 2 Graduate Institute of Medical Sciences, College of Medicine, Taipei Medical University, Taipei 110, Taiwan; 3 Department of Ecology and Environmental Sciences, Pondicherry University, Puducherry 605014, India; 4 Translational Medicine Center, Shin Kong Wu Ho-Su Memorial Hospital, Taipei 111, Taiwan; 5 Division of Cardiology, Department of Internal Medicine, Shuang Ho Hospital, Taipei Medical University, New Taipei City 235, Taiwan; 6 Department of Cardiovascular Center, Cathay General Hospital, Taipei 106, Taiwan; 7 School of Medicine, College of Medicine, Fu Jen Catholic University, New Taipei City 242, Taiwan

**Keywords:** glabridin, platelet, MAPK, microvascular thrombosis, PI3K/Akt/GSK3β, PLCγ2/PKC, pulmonary thromboembolism

## Abstract

Platelets are crucial for hemostasis and arterial thrombosis, which may lead to severe cardiovascular diseases (CVDs). Thus, therapeutic agents must be developed to prevent pathological platelet activation. Glabridin, a major bioalkaloid extracted from licorice root, improves metabolic abnormalities (i.e., obesity and diabetes) and protects against CVDs and neuronal disorders. To the best of our knowledge, no studies have focused on glabridin’s effects on platelet activation. Therefore, we investigated these effects in humans and mice. Glabridin exhibited the highest inhibitory effects on collagen-stimulated platelet aggregation and moderate effects on arachidonic-acid-stimulated activation; however, no effects were observed for any other agonists (e.g., thrombin or U46619). Glabridin evidently reduced P-selectin expression, ATP release, and intracellular Ca^2+^ ([Ca^2+^]i) mobilization and thromboxane A_2_ formation; it further reduced the activation of phospholipase C (PLC)γ2/protein kinase C (PKC), phosphoinositide 3-kinase (PI3K)/Akt/glycogen synthase kinase-3β (GSK3β), mitogen-activated protein kinase (MAPK), and NF-κB. In mice, glabridin reduced the mortality rate caused by acute pulmonary thromboembolism without altering bleeding time. Thus, glabridin effectively inhibits the PLCγ2/PKC cascade and prevents the activation of the PI3K/Akt/GSK3β and MAPK pathways; this leads to a reduction in [Ca^2+^]i mobilization, which eventually inhibits platelet aggregation. Therefore, glabridin may be a promising therapeutic agent for thromboembolic disorders.

## 1. Introduction

Cardiovascular diseases (CVDs) are the principal reason of death globally, and the rate of mortality has been increasing. The most common manifestations of CVDs are suggested to be thrombotic complications, such as ischemic stroke, venous thromboembolism, myocardial infarction, and peripheral artery diseases [1]. Platelets—anucleated blood cells—are released from megakaryocytes and play a key role in the development of CVDs [2]. Studies have shown that platelet activity varies across populations, which explains the variety of CVDs [3]. Following endothelial damage in blood vessels, platelets adhere to the damaged surface area, and subsequently release various biologically active constituents (e.g., thromboxane A_2_ (TxA_2_), ADP, and serotonin) that trigger platelet aggregation, which is considered to be the initiation of intraluminal thrombosis [4]. Platelet activation is generally stimulated by collagen or other soluble platelet agonists (e.g., ADP and arachidonic acid (AA)). Antiplatelet drugs inhibit the over-activation of platelets to prevent vascular thrombotic diseases [1]. However, the available antiplatelet drugs often lead to excessive bleeding, which warrants the development of highly effective and safe therapeutic agents for the inhibition of platelet activation.

**Figure 1:**
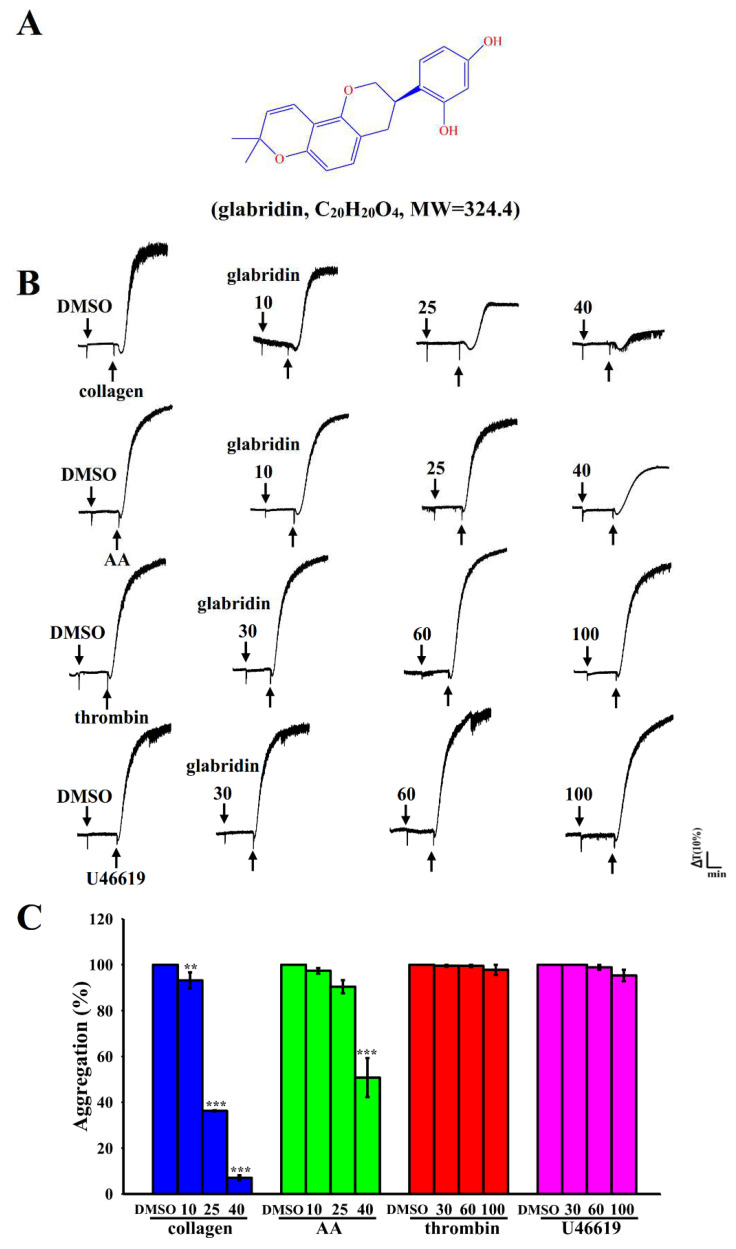
Inhibitory effects of glabridin on platelet aggregation stimulated by different agonists in human platelets. (**A**) Chemical structure of glabridin (C_20_H_20_O_4_). (**B**) Washed human platelets (3.6 × 10^8^ cells/mL) were preincubated with a solvent control (0.1% DMSO) or glabridin (10–100 μM) and subsequently treated with collagen (1 μg/mL), thrombin (0.01 U/mL), U46619 (1 μM), or arachidonic acid (AA; 60 μM) to stimulate platelet aggregation. (**C**) Concentration–response histograms of glabridin’s effects on platelet aggregation triggered by various agonists (%). Data are presented as mean ± standard error of the mean (n = 4). ** *p* < 0.01 and *** *p* < 0.001 vs. 0.1% DMSO-treated group.

The root of licorice, *Glycyrrhiza glabra* Linne, is a widely used herbal medicine worldwide; it is used to treat gastritis, bronchial and urological diseases, and food poisoning in Western and Asian countries [5]. In almost 50% of traditional Chinese herbal formulas, licorice is used (as a *guide drug*) with other herbs in a single prescription to enhance the efficacy of the other herbs, reduce toxicity, and improve flavor [6]. Licorice contains several bioactive components, including glycyrrhizic acid, glycyrrhetinic acid, isoliquiritigenin, licochalcone, and glabridin [7]. Glabridin (4-[(3*R*)-8,8-dimethyl-3,4-dihydro-2*H*-pyrano[2,3-f] chromen-3-yl]benzene-1,3-diol; Figure 1A), a polyphenolic flavonoid, is a major constituent, making up about 0.2% in the root of licorice [8]. Glabridin has been shown to have pharmacological value in improving metabolic abnormalities (i.e., obesity, diabetes, and CVDs); protecting the nervous system; functioning as a substitute for estrogen; preventing infections caused by *Staphylococcus* sp., *Candida* sp., and other bacteria; and functioning as an anti-cancer, anti-inflammatory, and anti-osteoporotic agent [9]. Clinical studies have suggested that glabridin reduces the levels of lipids and glucose in the blood of patients with overweight or diabetes. Carmeli et al. [10] reported that the dietary supplementation of a 60 mg glabridin containing glycyrrhizin-free licorice root extract reduced low-density lipoprotein (LDL oxidation) in the plasma of healthy individuals. Collectively, these findings suggest that glabridin inhibits LDL oxidation both in vitro and in vivo; thus, glabridin might be a promising drug candidate for the treatment of atherosclerosis or CVDs.

Licochalcone, a chalcone derivative from licorice, has been reported to reduce rabbit and rat platelet activation via the inhibition of cyclooxygenase-1 activity [11]. In addition, Lien et al. [12] also reported the inhibitory effects of licochalcone in human platelets through PLCγ2–PKC and MAPK signaling mechanisms, and also how it prevents thrombus formation in animal models. However, despite the various pharmacological activities of glabridin, to our knowledge, there are no studies were conducted to evaluate its effects on platelet activation. Thus, the present study is the first to systemically investigate the possible inhibitory effects of glabridin on platelet activation. Both humans (ex vivo) and mouse models of experimental thrombosis (in vivo) were used in the present study.

## 2. Results

### 2.1. Effects of Glabridin on Platelet Aggregation Stimulated by Various Agonists in Humans

At concentrations of 10–40 µM, glabridin exhibited the highest inhibitory effects on collagen (1 μg/mL)-stimulated human platelet aggregation and moderate inhibitory effects on AA (60 µM)-stimulation (Figure 1B,C). However, it exerted no prominent effects on platelet inhibition stimulated by thrombin (0.01 U/mL) or U46619 (1 µM; an analog of endoperoxide prostaglandin), even at high concentrations of 60–100 μM (Figure 1C). These outcomes specified that the efficacy of glabridin varies across platelet agonists. The approximate IC_50_ (25 µM) and maximal (40 µM) concentrations of glabridin were subsequently used to elucidate the possible mechanisms underlying the effects of glabridin on collagen-stimulated platelet activation. The solvent control (0.1% DMSO) did not exert any significant effects on platelet aggregation (Figure 1B). Moreover, there are no differences in platelet aggregation when comparing collagen alone (with Tyrode’s solution) with collagen plus 0.1% DMSO groups (Figure S1).

### 2.2. Effects of Glabridin on ATP Release, Relative Intracellular Ca^2+^ Change, and P-Selectin Surface Expression and TxB_2_ Formation

**Figure 2:**
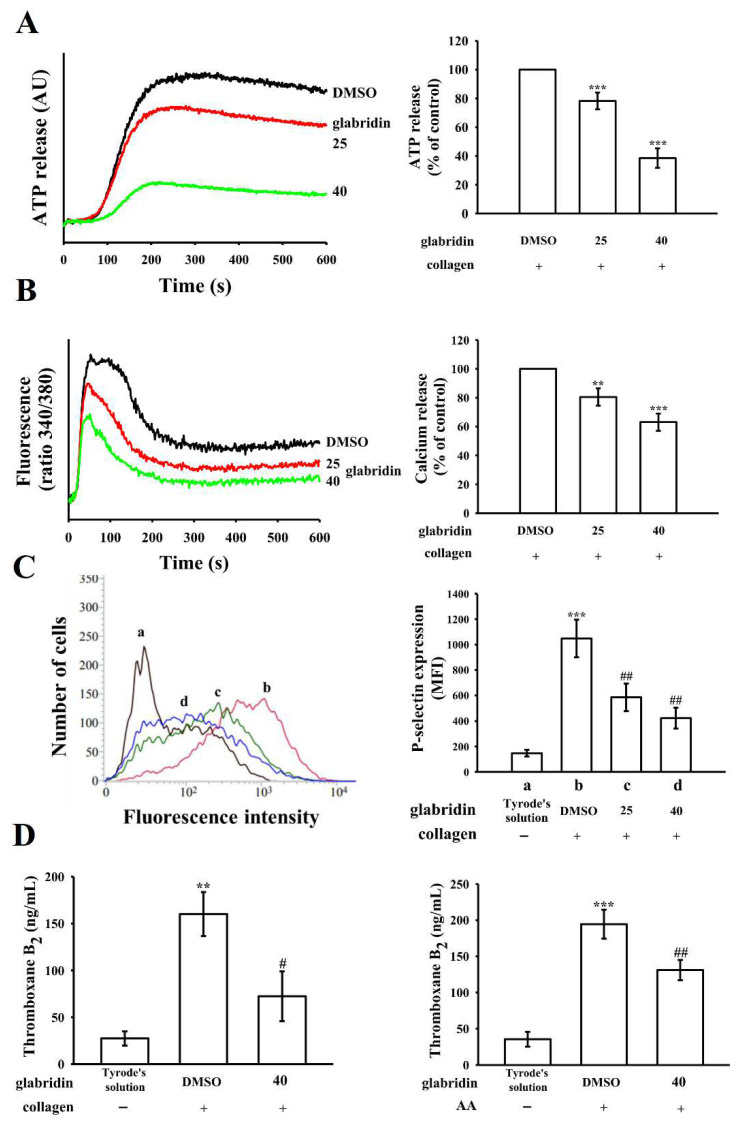
Inhibitory effects of glabridin on ATP release, relative [Ca^2+^]i mobilization, P-selectin surface expression, and thromboxane B_2_ formation in human platelets. Washed platelets (3.6 × 10^8^ cells/mL) were preincubated with DMSO (0.1%) or glabridin (25 and 40 µM), followed by the addition of collagen (1 μg/mL) or arachidonic acid (AA; 60 μM) to trigger (**A**) ATP release (arbitrary unit [AU]), (**B**) relative [Ca^2+^]i mobilization, and (**C**) P-selectin surface expression (mean fluorescence intensity [MFI]) ((a) Tyrode’s solution, (b) collagen-stimulated platelets, (c) glabridin 25 µM, and (d) 40 µM). (**D**) Thromboxane B_2_ (TxB_2_) formation. Respective statistical analyses are indicated in the bar diagrams. Data are presented as the mean ± standard error of the mean (n = 4). (**A**,**B**) ** *p* < 0.01 and *** *p* < 0.001 vs. 0.1% DMSO + collagen group. (**C**,**D**) *** *p* < 0.001 vs. resting platelets (in Tyrode’s solution); # *p* < 0.05 and ## *p* < 0.01 vs. 0.1% DMSO + collagen group.

The release of the platelet granules contents (e.g., P-selectin, ADP/ATP, and Ca^2+^) is highly associated with platelet activation. Glabridin markedly inhibited the release of ATP from collagen-stimulated platelets (Figure 2A). Increasing the levels of intracellular Ca^2+^ ([Ca^2+^]i) leads to platelet aggregation. At concentrations of 25 and 40 μM, glabridin noticeably reduced [Ca^2+^]i upsurge by nearly 20% and 37%, respectively, compared with the 0.1% DMSO control (Figure 2B). P-selectin is a key biomarker for platelet activation. Under resting condition (in Tyrode’s solution), P-selectin is normally expressed on the inner walls of α-granules; upon activation, platelets expose the inner walls of these granules to the outer parts of the cells [13]. As shown in Figure 2C, glabridin reduced the collagen-stimulated surface expression of P-selectin; the related statistical data are presented on the right-hand side of the figure: (a) resting control: 148 ± 26, (b) collagen-stimulated platelets: 1048 ± 147, (c) 25 μM glabridin: 586 ± 106, and (d) 40 μM glabridin: 423 ± 82; n = 4. In Figure 2D, resting platelets produced relatively little TxB_2_ (27 ± 8 ng/mL and 36 ± 10 ng/mL; n = 4) compared with collagen- (160 ± 23 ng/mL; n = 4) or AA- (194 ± 20 ng/mL; n = 4) stimulated platelets. Glabridin (40 μM) markedly reduced TxB_2_ formation stimulated by collagen (72 ± 26 ng/mL; n = 4) and AA (131 ± 14 ng/mL; n = 4), respectively.

### 2.3. Characteristics of Glabridin on Phospholipase Cγ2/Protein Kinase C Activation

**Figure 3:**
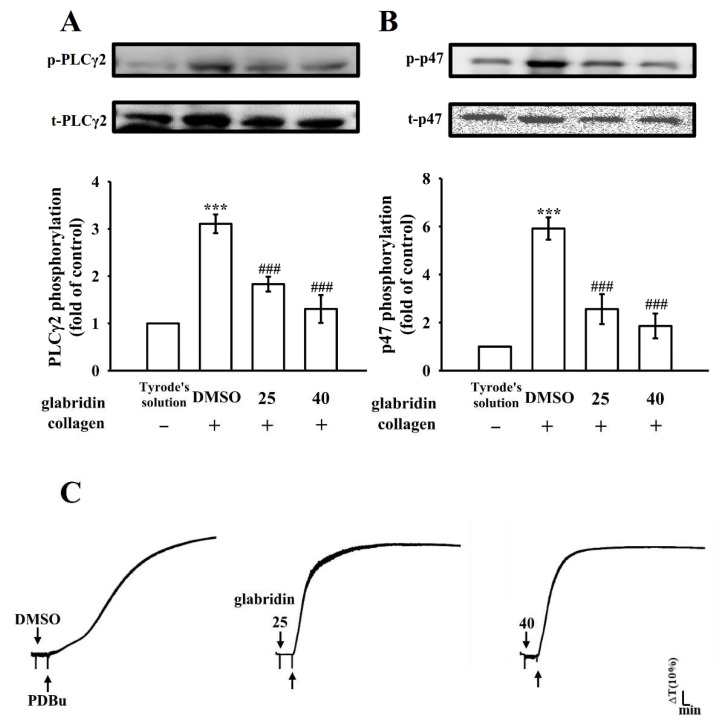
Effects of glabridin on the activation of phospholipase Cγ2 and protein kinase C in platelets. DMSO (0.1%) or glabridin (25 and 40 µM) were preincubated in washed platelets, and they were subsequently treated with collagen (1 µg/mL) or phorbol 12,13-dibutyrate (PDBu, 150 nM) to stimulate (**A**) phospholipase Cγ2 (PLCγ2) activation, (**B**) protein kinase C (PKC) activation (p–p47), or (**C**) platelet aggregation. Data are shown as the mean ± standard error of the mean (n = 4). *** *p* < 0.001 vs. resting platelets (in Tyrode’s solution); ### *p* < 0.001 vs. 0.1% DMSO + collagen group. Diagram in (**C**) represents four independent experiments.

Phospholipase C (PLC) hydrolyzes phosphatidylinositol 4,5-bisphosphate to produce diacylglycerol (DAG) and inositol trisphosphate (IP_3_), the two main secondary messengers. DAG stimulates protein kinase C (PKC), triggering the activation of a nearly 47-kDa protein that is primarily phosphorylated (pleckstrin or p47), thus leading to the secretion of granules; IP_3_ elevates calcium influx [14]. In the present study, glabridin (25 and 40 µM) reduced both PLCγ2 phosphorylation and PKC activation in collagen-stimulated platelets (Figure 3A,B). However, neither 25 nor 40 µM glabridin considerably affected platelet aggregation stimulated by phorbol 12,13-dibutyrate (PDBu, a PKC activator; Figure 3C); this suggested that glabridin did not exert its direct effects on PKC, but on PLCγ2. Moreover, there are no differences in PKC activation between the collagen alone (with Tyrode’s solution) and collagen with 0.1% DMSO groups (Figure S2).

### 2.4. Glabridin on Phosphoinositide 3-Kinase/Akt/Glycogen Synthase Kinase-3β and Mitogen-Activated Protein Kinases Activation

**Figure 4:**
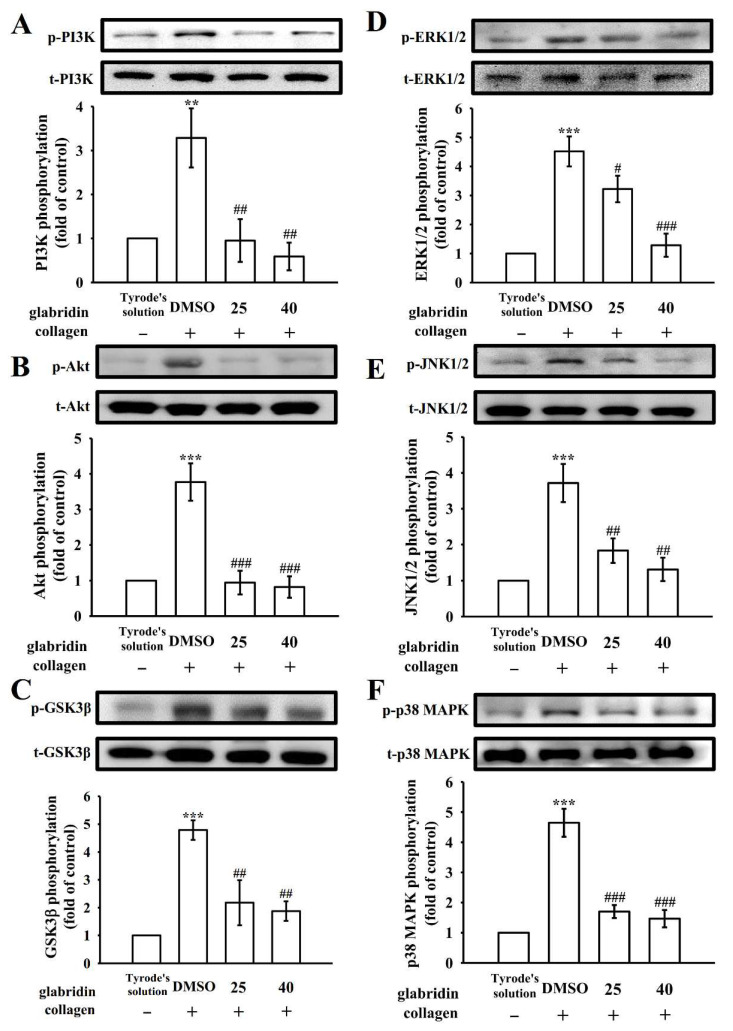
Regulatory activity of glabridin on phosphoinositide 3-kinase (PI3K)/Akt/glycogen synthase kinase-3β (GSK3β) and mitogen-activated protein kinase (MAPK) pathways in platelets. Washed platelets were preincubated with DMSO (0.1%) or glabridin (25 and 40 µM) and treated with collagen (1 μg/mL) for the immunoblotting of (**A**) PI3K, (**B**) Akt, (**C**) GSK3β, (**D**) ERK1/2, (**E**) JNK1/2, and (**F**) p38 MAPK. Data are shown as the mean ± standard error of the mean (n = 4). ** *p* < 0.01 and *** *p* < 0.001 vs. resting platelets (in Tyrode’s solution); # *p* < 0.05, ## *p* < 0.01, and ### *p* < 0.001 vs. 0.1% DMSO + collagen group.

Under the high shear stress, the phosphoinositide 3-kinase (PI3K)/Akt/glycogen synthase kinase-3β (GSK3β) pathway is involved in thrombus formation [15]. PI3K substantially plays role in platelet activation, and functions as the primary regulator of Akt activation [15]. The Akt (also known as protein kinase B) pathway is involved in cell growth and survival, and can be activated by several platelet agonists that control platelet activation and hemostasis. GSK3β is a classical enzyme regulated downstream of the PI3K/Akt pathway in platelets [16]. In the present study, glabridin (25 and 40 μM) markedly inhibited the PI3K/Akt/GSK3β pathway in collagen-stimulated platelets (Figure 4A–C). The mitogen-activated protein kinases (MAPKs) pathway is involved in various cellular functions, such as cell proliferation, apoptosis, inflammation, and platelet activation. In platelets, the MAPKs pathway mainly contains extracellular signal-regulated kinase (ERK)1/2, Jun N-terminal kinase (JNK)1/2, and p38 MAPK [17]. In the present study, glabridin (25 and 40 μM) inhibited the collagen-stimulated phosphorylation of all three aforementioned MAPKs, indicating that MAPKs signaling is involved in the glabridin-mediated inhibition of platelet activation (Figure 4D–F). Together, these results substantiate that the inhibition of both PI3K/Akt/GSK3β and MAPK pathways contribute to the glabridin-mediated inhibition of platelet activation.

### 2.5. Effects of Glabridin on NF-κB Signaling

**Figure 5:**
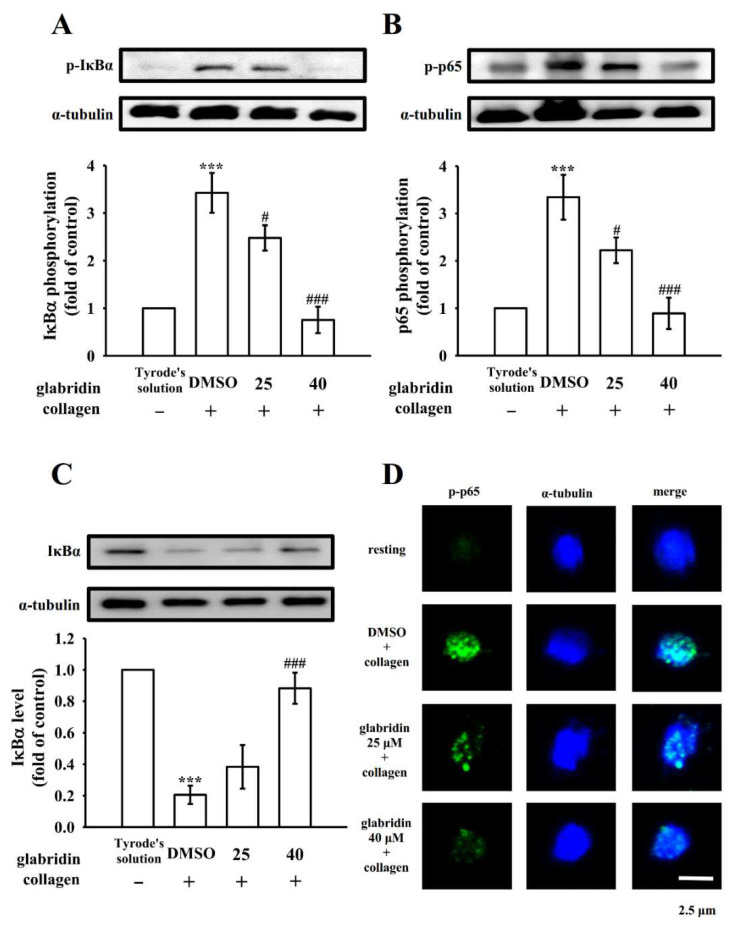
Effects of glabridin on the activation of NF-κB in human platelets. Washed platelets were preincubated with DMSO (0.1%) or glabridin (25 and 40 µM) and then treated with collagen (1 μg/mL) for the immunoblotting of (**A**) IκBα and (**B**) p65 phosphorylation, or (**C**) IκBα degradation, and confocal microscopic assessment (1000× magnification) of (**D**) phosphorylated NF-κB (p65) (green fluorescence) and α-tubulin (blue fluorescence) using goat anti-rabbit CF^TM^ 488A and anti-mouse CF^TM^ 405M dyes, respectively. Data are expressed as the mean ± standard error of the mean (n = 4). *** *p* < 0.001 vs. resting platelets (in Tyrode’s solution); # *p* < 0.05 and ### *p* < 0.001 vs. 0.1% DMSO + collagen group. The confocal images represent four independent experiments. Bar: 2.5 μm.

Pleiotropic NF-κB normally exists as an inactive cytoplasmic complex, and its predominant form is a heterodimer comprising p50 and p65 subunits tightly bound to the inhibitory proteins of the IκB family [18]. The phosphorylation of both IκBα and p65 and the degradation of IκBα considerably increased after the stimulation of platelets with collagen (1 μg/mL); glabridin (25 and 40 μM) reduced the phosphorylation of IκBα and p65 (Figure 5A,B) and reversed the degradation of IκBα (Figure 5C). The inhibitory effects of glabridin on NF-κB activation were further confirmed using confocal laser scanning fluorescence microscopy, which exhibited green fluorescence (p65 activation) and blue fluorescence (α-tubulin) in resting or collagen-stimulated platelets. Collagen (1 µg/mL) increased the fluorescent brightness of phosphorylated p65 (p-p65) compared with that observed in resting platelets; the intensity was reduced in glabridin-treated platelets (Figure 5D). However, no significant differences in α-tubulin intensity were noted between the groups (Figure 5D). These results suggest that the inhibition of NF-κB activation is crucial for the glabridin-mediated inhibition of platelet activation. 

### 2.6. Activity of Glabridin on Experimental Acute Pulmonary Thrombosis and Bleeding Time

**Figure 6:**
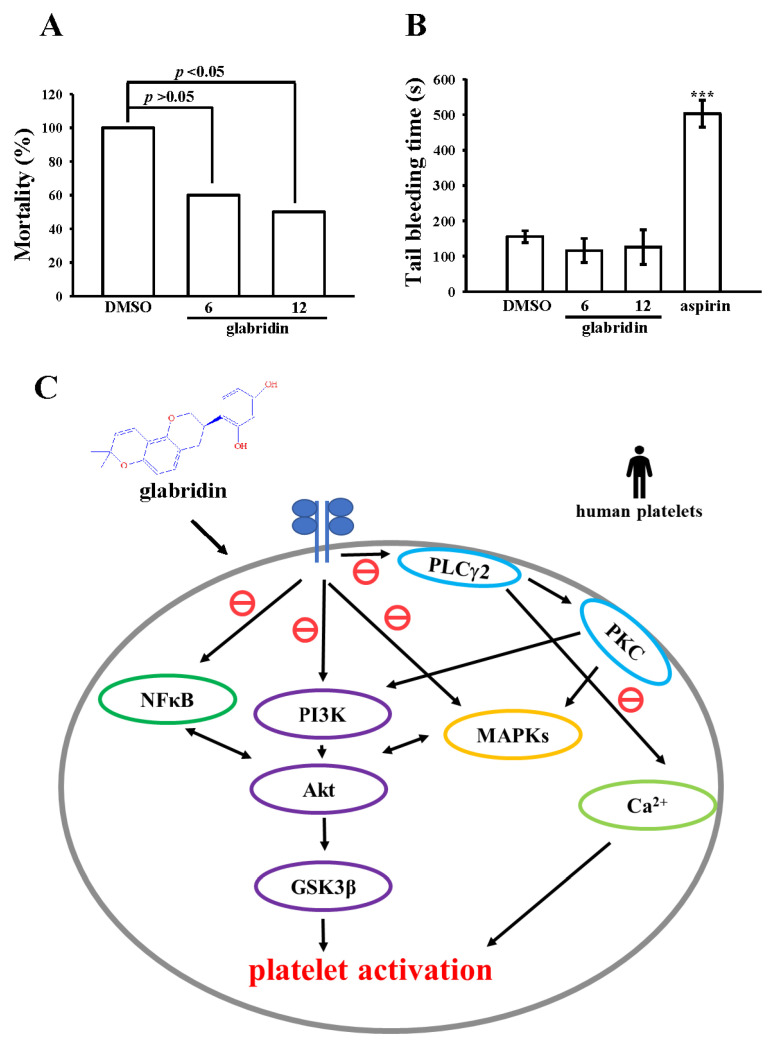
Effects of glabridin on the degree of acute pulmonary thromboembolism and time of tail vein bleeding. (**A**) The development of acute pulmonary thrombosis in mice was induced by ADP (700 mg/kg) through tail veins injection after they were treated DMSO (0.1%) or glabridin (6 and 12 mg/kg) through intraperitoneal route. (**B**) Bleeding time was measured via the tail vein transection model after 30 min of the intraperitoneal administration of DMSO (0.1%), glabridin (6 and 12 mg/kg), or aspirin (1 mg/kg). Data are expressed as the mean ± standard error of the mean (n = 10). *** *p* < 0.001 vs. 0.1% DMSO-treated group. Data in (**A**) are presented as mortality rates. (**C**) Proposed scheme of the mechanisms underlying the inhibitory effects of glabridin on platelet activation in humans. Glabridin inhibits platelet activation associated with signaling cascades (e.g., PLCγ2/PKC, PI3K-Akt-GSK3β, and MAPKs), followed by the regulation of [Ca^2+^]i mobilization, which eventually inhibits platelet aggregation.

The therapeutic effects on the antithrombotic activity of glabridin were evaluated in this study. Glabridin reduced the mortality rates of mice with ADP-induced acute pulmonary embolism (Figure 6A). The results substantiated that glabridin at concentrations of 6 and 12 mg/kg considerably reduced the rates of ADP (700 mg/kg)-induced mortality from 100% (10 dead, n = 10; 0.1% DMSO-treated control) to 60% (six dead, n = 10; *p* > 0.05) and 50% (five dead, n = 10; *p* < 0.05), respectively. Furthermore, we investigated the bleeding time through the tail vein transection bleeding approach 30 min after the intraperitoneal administration of glabridin and aspirin; the bleeding times (n = 10) were 155 ± 16, 116 ± 34, 126 ± 49, and 503 ± 38 s for 0.1% DMSO-treated, 6 mg/kg glabridin–treated, 12 mg/kg glabridin–treated, and 1 mg/kg aspirin–treated groups (Figure 6B). To check if there was any rebleeding, though the bleeding had stopped, the mice were separately observed for 15 min. The results advocated that compared with aspirin, glabridin considerably abridged the degree of pulmonary thromboembolism without substantially prolonging the bleeding time.

## 3. Discussion

The administration of bioactive alkaloids exerts antiatherogenic and antithrombotic effects on patients with CVDs [19]. Glabridin appears to exert its biological effects by affecting multiple targets. Regarding cardiovascular protection, the inhibition of LDL oxidation, macrophage activation, and adhesion molecule expression by glabridin may synergistically contribute to its beneficial effects [20]. The protection conferred by glabridin against CVDs has been demonstrated in animal and clinical studies, suggesting glabridin is a suitable candidate for treating CVDs, or a promising adjuvant for improving the pharmacokinetic characteristics of other medicines. A pharmacokinetic study demonstrated that glabridin could readily enter the human body due to its easy absorption; in rats, after the oral administration of glabridin at concentrations of 5 and 20 mg/kg, the Cmax of glabridin was 15.10 ± 4.72 and 60.41 ± 18.87 ng/mL, respectively [21]. Although the amount of regular glabridin acquired from natural sources is insufficient to attain the concentration required to inhibit platelet activation in vivo, its long-term consumption, particularly through Chinese medicines with long-term therapeutic applications, is ideal for preventing atherothrombotic events.

Platelet activation is usually associated with a series of phosphorylation of tyrosine kinases, which leads to an increase of [Ca^2+^]i and granule secretion (i.e., P-selectin and ATP) from platelets. The protein storage compartment of platelets mostly contains α-granules. It also contains membrane-associated (e.g., P-selectin) and soluble (e.g., fibrinogen and platelet-derived growth factor) proteins. The exocytosis of α-granules is a marker of platelet activation, which is estimated on the basis of P-selectin expression that can be measured using flow cytometry. TxB_2_ formation, a stable metabolite of TxA_2_, was markedly inhibited by glabridin. TxA_2_ is a relatively strong platelet agonist that can lead to aggregate formation. Phosphoinositide breakdown can induce TxA_2_ formation via free AA release by diglyceride lipase or by endogenous phospholipase A_2_ (PLA_2_) produced from membrane phospholipids [22]. TxA_2_ interacts with and activates the platelet thromboxane receptor; thus, multiple intracellular responses, including intracellular Ca^2+^ release, can be observed [22]. Therefore, TxA_2_ is important for collagen and AA-induced platelet aggregation, and this may explain glabridin’s strong inhibitory activity in platelet aggregation stimulated by collagen or AA. Additionally, glabridin effectively inhibited collagen-stimulated platelet aggregation, which suggests that it mediated one of the important signals of PLC-dependent mechanism. Platelet activation influences the stimulation of phospholipases, particularly PLC, which leads to the formation of IP_3_ and DAG, which, in turn, activates PKC and subsequently induces the phosphorylation of p47 [23]. The PLCγ family comprises isozymes 1 and 2, and PLCγ2 participates in collagen-dependent signaling in platelets [24]. In the present study, glabridin inhibited collagen-stimulated PLCγ2/PKC activation; however, it might not have directly affected PKC activation because PDBu-induced platelet aggregation remained unaffected. This result proposes that PLCγ2 downstream signaling may play a vital role in the glabridin-mediated inhibition of platelet activation.

MAPK cascades are key signaling pathways that control various cellular events, including proliferation, differentiation, and apoptosis. The results from the MAPK-specific inhibitors or knockout mice indicates the presence of ERK1/2, JNK1/2, and p38 MAPK in platelets and their role in platelet activation [25]; although, one study suggested that JNK1/2 and ERK1/2 suppress the activation of integrin α_IIb_β_3_ [26]. However, their unique roles in platelets remain unclear. Another study concluded that the activation of ERK is essential in collagen-stimulated platelet aggregation [27]. Cytosolic phospholipase A_2_ catalyzes the release of AA to produce thromboxane A_2_, which is a key substrate for the activation of p38 MAPK by various platelet agonists [27]. The present study revealed that glabridin substantially inhibited the activation of ERK1/2 and JNK1/2 or p38 MAPK; this may be the reason for the higher efficacy of glabridin in inhibiting collagen- or AA-stimulated platelet activation than in inhibiting U46619- or thrombin-stimulated platelet activation.

PI3K activation strongly contributes to platelet activation. It acts downstream of several platelet receptors, including the collagen receptor, glycoprotein (GP) VI, which regulates PLCγ2 activation and Ca^2+^ mobilization [28] or the ADP receptors, P2Y12 and integrin α_IIb_β_3_ [29]. Akt is a key and universal effector of PI3K. Mice lacking Akt exhibit impaired platelet aggregation and stable adhesion under flow [30]. Hence, protein kinase-mediated activation of Akt, particularly by PI3K, may be an attractive target for antithrombotic drugs. PI3K/Akt and MAPKs are mutually activated in platelets, and PKC is their upstream regulator (Figure 6C) [31]. Whether the downstream signaling of Akt is involved in platelet activation remains unknown; several candidates, such as GSK3 (α and β isoforms), have been identified and expressed in platelets, and GSK3β is the most abundant protein [32,33]. The inhibition of GSK3 appears to be necessary for thorough platelet activation by various agonists. PI3Kβ knockout mice exhibited arterial thrombus instability while they were under high shear stress because of impaired Akt/GSK3 activation within the growing thrombus [15]. However, the mechanism of GSK3-mediated platelet activation remains obscure. Thus, the identification of the GSK3 substrates in the platelet may help determine auspicious candidates for the development of novel drugs for antithrombotic diseases. On the whole, the PI3K/Akt/GSK3β signaling cascade seems to have a key role in platelet activation and thrombus formation and stability under high shear stress in vivo.

Activated NF-κB in human atherosclerotic plaques leads to the development of unstable coronary plaques [34]. Although platelets lack nuclei, they contain several functional transcription factors and NF-κB. NF-κB is reportedly involved in platelet activation, including IKKβ phosphorylation, IκBα degradation, and p65 phosphorylation [35,36], which suggests that NF-κB plays crucial roles in platelet activation apart from those genomic functions. Immunoblotting and confocal microscopy assays in the present study demonstrated that NF-κB activation induced by collagen was potently inhibited by glabridin in human platelets; this indicates that NF-κB signaling plays a specific role in the glabridin-mediated inhibition of platelet activation. NF-κB inhibitors have been reported to suppress platelet activation [37] and exhibit novel cooperative activity with the PI3K/Akt pathway after platelet activation (Figure 6C) [38]. Furthermore, Lien et al. [12] found that licochalcone’s inhibitory effect against human platelet aggregation stimulated by collagen, thrombin, and U46619, and its mechanisms may be mediated by blocking integrin α_IIb_β_3_, PLC-PKC, and MAPK activation. However, our results show that glabridin markedly inhibits human platelet aggregation stimulated by collagen and AA, but not by thrombin and U46619; its mechanisms were suggested, at least partly, by impeding the PI3K/Akt/GSK3β and NF-κB pathway. Therefore, licorice containing at least two alkaloids, licochalcone and glabridin, can effectively diminish platelet activation in humans.

To further evaluate the therapeutic efficacy of the test compound against vascular thrombosis, animal experiments were performed in the present study. Momi et al. [39] reported that the intravenous injection of collagen with epinephrine markedly induced platelet pulmonary thromboembolism in mice, which resulted in a dose-dependent increase of lung vessels occluded by platelet thromboemboli, and a marked drop of the number of circulating platelets. The histological analysis of lungs revealed that a substantially high number of lung vessels were completely or partially occluded by platelet thrombi after injection [39]. Glabridin effectively reduced the rate of mortality associated with acute pulmonary thromboembolism without altering bleeding time, unlike aspirin, which increased bleeding time. Therefore, glabridin is a valuable natural compound that may be used to treat thromboembolic-related disorders.

## 4. Materials and Methods

### 4.1. Chemicals and Reagents

Glabridin (≥98%), collagen (type I), bovine serum albumin (BSA), aspirin, heparin, ethylenediaminetetraacetate (EDTA), luciferin–luciferase, AA, 9,11-dideoxy-11α,9α-epoxymethanoprostaglandin (U46619), prostaglandin E_1_, phenylmethylsulfonyl fluoride, sodium orthovanadate, sodium pyrophosphate, aprotinin, leupeptin, sodium fluoride, PDBu, paraformaldehyde, and thrombin were purchased from Sigma (St. Louis, MO, USA). The TxB_2_ enzyme-linked immunosorbent assay (ELISA) kit was purchased from Cayman Chemical (Ann Arbor, MI, USA). The anti-phospho-p38 MAPK (Thr^180^/Tyr^182^) polyclonal antibody (pAb) was purchased from Affinity (Cincinnati, OH, USA). Anti-phospho-JNK (Thr^183^/Tyr^185^), anti-phospho-p44/p42 ERK (Thr^202^/Tyr^204^), anti-phospho-(Ser) PKC substrate, and anti-phospho-PI3 kinase p85 (Ty^r458^)/p55 (Tyr^199^) pAbs and anti-Akt, anti-p38 MAPK, anti-PLCγ2, and anti-PI3K p85 (19H8) monoclonal antibodies (mAbs) were purchased from Cell Signaling (Beverly, MA, USA). The anti-phospho PLCγ2 mAb was obtained from Abcam (Cambridge, UK). Anti-phospho-GSK3α/β and anti-GSK3α/β mAbs were purchased from Santa Cruz Biotechnology (Santa Cruz, CA, USA). Anti-IκBα (44D4) and anti-phospho-IκBα (Ser^32^/^36^) (5A5) mAbs and anti-phospho-NF-κB p65 (Ser^536^) pAb were purchased from Cell Signaling (Beverly, MA, USA). The protein assay dye reagent concentrate was purchased from Bio-Rad Laboratories (Hercules, CA, USA), and anti-phospho-Akt (Ser^473^) pAb was purchased from BioVision (Mountain View, CA, USA). Fura 2-AM was obtained from Molecular Probes (Eugene, OR, USA). FITC-anti-human CD42P (P-selectin) mAb was purchased from BioLegend (San Diego, CA, USA). CF^TM^488A Dye and CF^TM^405M Dye were obtained from Biotium (Hayward, CA, USA). Anti-α-tubulin mAb was purchased from NeoMarkers (Fremont, CA, USA). Hybond-P polyvinylidene difluoride membranes, enhanced chemiluminescence western blotting detection reagent, horseradish peroxidase-conjugated donkey anti-rabbit immunoglobulin G (IgG), and sheep anti-mouse IgG were obtained from Amersham (Buckinghamshire, UK). Glabridin was dissolved in 0.1% DMSO and stored at 4 °C for the experiments.

### 4.2. Human Platelet Preparation and Aggregation

This study was conducted in accordance with the ethical principles of the Declaration of Helsinki and was approved by the Institutional Review Board of Taipei Medical University (TMU-JIRB-N201812024). All human participants provided informed consent for participation. Washed human platelets (3.6 × 10^8^ cells/mL) were prepared using the samples obtained from a total of 30 healthy human participants, following the methods described in a previous study [40]. In brief, blood samples were subjected to centrifuge after mixing with acid/citrate/glucose (9:1, *v*/*v*). The collected supernatant (PRP) was incubated with EDTA (2 mM) and heparin (6.4 U/mL) for 5 min, and again centrifuged at 500× *g* for 10 min. The platelet-containing pellet was resuspended in 5 mL Tyrode’s solution for 10 min at 37 °C. After centrifugation, the washing process was repeated and finally suspended in Tyrode’s solution containing BSA (3.5 mg/mL). Platelets were counted using a Coulter counter (Beckman Coulter, Miami, FL, USA). The final concentration of Ca^2+^ in Tyrode’s solution was 1 mM. The solvent control (0.1% DMSO) and glabridin (10–100 μM) were preincubated with platelets for 3 min before stimulation with collagen (1 μg/mL), thrombin (0.01 U/mL), U46619 (1 μM), and AA (60 μM). Platelet aggregation was measured using a Lumi-Aggregometer (Payton, Scarborough, ON, Canada), and a turbidimetric method was used for measurements [41]. The degree of platelet aggregation was measured as a percentage of the platelet aggregation observed in the control (the group treated with Tyrode’s solution) in light transmission units. In addition, the level of ATP release was tested in accordance with the manufacturer’s instructions (Hitachi Spectrometer F-7000 (Tokyo, Japan)).

### 4.3. Change of [Ca^2+^]i and P-Selectin Surface Expression in Human Platelets

To measure ([Ca^2+^]i) mobilization, citrated whole blood was centrifuged, and the supernatant was incubated with Fura 2-AM (5 μM), which was assessed using a Hitachi Spectrometer F-7000 (Tokyo, Japan) at excitation (340 and 380 nm) and emission (500 nm) wavelengths [41]. Washed platelets (3.6 × 10^8^ cells/mL) were preincubated with either 0.1% DMSO or glabridin (25 and 40 µM) and FITC-conjugated anti-P-selectin mAb (2 µg/mL) for 3 min, followed by stimulation with collagen (1 µg/mL), and the final suspensions were used to examine fluorescein-labeled platelets using a flow cytometer (FAC Scan system; Becton Dickinson, San Jose, CA, USA). Data were collected from a total of 50,000 platelets in each group, and the platelets were identified on the basis of their characteristic forward and orthogonal light-scattering profiles. All experiments were performed at least four times to ensure reproducibility.

### 4.4. Measurement of TxB_2_ Formation

Platelet suspensions (3.6 × 10^8^ cells/mL) were preincubated with 0.1% DMSO or glabridin (40 µM) for 3 min, followed by the addition of collagen (1 µg/mL) or AA (60 µM). Six minutes after the addition of agonists, EDTA (2 mM) and indomethacin (50 µM) were added to the suspensions and centrifuged at 2000× *g* for 5 min. Finally, the TxB_2_ levels were measured from the supernatants using an ELISA kit according to the manufacturer’s instructions.

### 4.5. Immunoblotting

Washed platelets at a density of 1.2 × 10^9^ cells/mL were preincubated with solvent control (0.1% DMSO) and glabridin (25 and 40 µM), followed by collagen (1 μg/mL) activation for 5 min or no activation. Later, the platelets were directly resuspended in 200-μL lysis buffer (10 μg/mL aprotinin, 1 mM PMSF, 2 μg/mL leupeptin, 10 mM NaF, 1 mM sodium orthovanadate, and 5 mM sodium pyrophosphate) and centrifuged at 5000× *g* for 5 min. After centrifugation of the lysates, the supernatant was collected, and 80 μg of protein was separated from the supernatant through sodium dodecyl sulfate–polyacrylamide gel (12%) electrophoresis. A Bradford protein assay (Bio-Rad, Hercules, CA, USA) was performed to quantitate protein concentrations. The proteins of interest were spotted using their respective primary antibodies. The intensity of protein bands was measured using a video densitometer and the Bio-Profil Biolight software (version V2000.01; Vilber Lourmat, Marne-la-Vallée, France). Relative protein expression was calculated after normalization to the total protein of interest.

### 4.6. Confocal Laser Scanning Fluorescence Microscopy

Platelets were immunostained as per the method described previously [42]. Briefly, resting or collagen (1 μg/mL)-stimulated platelets (1.2 × 10^9^ cells/mL) were fixed in 4% (*v*/*v*) paraformaldehyde on poly-l-lysine-coated coverslips for 1 h. Platelets were then permeabilized in 0.1% Triton X-100 and incubated with 5% BSA in phosphate-buffered saline (PBS) for 1 h before staining. To observe p-p65 and α-tubulin, platelets were stained with anti-phospho-NF-κB p65 (Ser^536^) pAb and α-tubulin mAb for 24 h. After washing with PBS, the platelets were further incubated with goat anti-rabbit CF^TM^488A dye or goat anti-mouse CF^TM^405M dye for 1 h, and then observed under a confocal microscope (Leica TCS SP5, Mannheim, Germany) using a 100× oil immersion objective lens.

### 4.7. ADP-Induced Acute Pulmonary Thromboembolism in Mice

Acute pulmonary microvascular thrombosis was induced following a method described in a previous study [43]. All procedures in this study were performed after obtaining approval from the Institutional Animal Care and Use Committee of Taipei Medical University (LAC-2021-0084). Male ICR mice were intraperitoneally injected with 50 μL of DMSO (0.1%) or glabridin (6 and 12 mg/kg). After 5 min, ADP (700 mg/kg) was injected into each mouse’s tail vein. Within 10 min after injection, the rate of mortality was calculated for each group.

### 4.8. Measurement of Tail Vein Bleeding Time

Bleeding time was measured through the tail vein transection bleeding approach. Briefly, mice were intraperitoneally administered 50 μL of DMSO (0.1%), glabridin (6 and 12 mg/kg), or aspirin (1 mg/kg), and they were anesthetized for 30 min. The tails were cut 3 mm from the tip with a scalpel, and immediately placed in a tube containing normal saline at 37 °C. The bleeding time was monitored until it stopped completely.

### 4.9. Statistical Analysis

The results of this study are expressed as the mean ± standard error of the mean. Values of n refer to the number of experiments performed using the samples of different blood donors. Significant differences among the experimental groups were analyzed using one-way analysis of variance with the Student–Newman–Keuls post hoc test to control for family-wise type I error. In addition, differences of pulmonary microvascular thrombosis in mice were assessed using Fisher’s exact test. The statistical significance was set at *p* < 0.05 using SAS (version 9.2; SAS Inc., Cary, NC, USA).

## 5. Conclusions

Glabridin effectively exhibits anti-platelet effects by impeding the PLCγ2–PKC cascade and successively prevents the activation of the PI3K/Akt/GSK3β and MAPK pathways; these events subsequently lead to the reduction of P-selectin expression, ATP release, and [Ca^2+^]i mobilization. Together, these events ultimately prevent platelet aggregation. The findings of this study may provide insights into the role of glabridin in the prevention of CVDs.

## Data Availability

The data presented in this study are available in the article.

## Supplementary Materials

The following supporting information can be downloaded at: https://www.mdpi.com/article/10.3390/ijms231911372/s1.
