# Supplementary material for: Glabridin, a Bioactive Flavonoid from Licorice, Effectively Inhibits Platelet Activation in Humans and Mice
Source: Int J Mol Sci. 2022 Sep 27;23(19):11372. doi: 10.3390/ijms231911372 (PMC9570097; doi:10.3390/ijms231911372)
Supplement: Supplementary file 1 [file ijms-23-11372-s001.zip › ijms-1885095-supplementary.pdf]

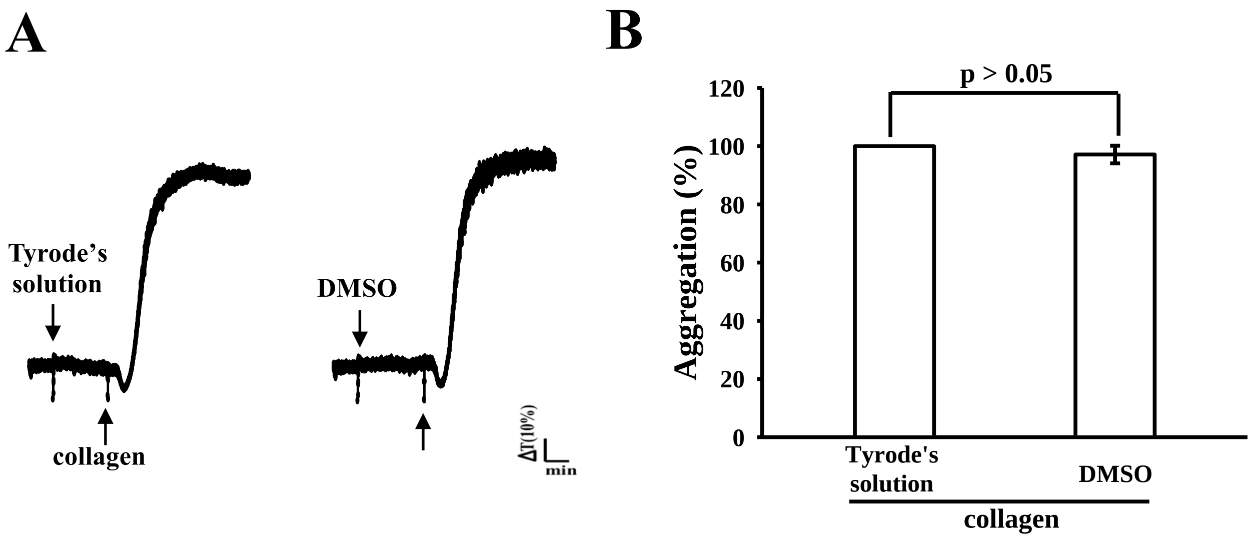

Figure S1. Aggregation activity on collagen-induced platelet aggregation. (A) Washed human platelets (3.6×10^8 cells/mL) were preincubated with Tyrode’s solution or 0.1% DMSO and subsequently treated with collagen (1 μg/mL) to stimulate platelet aggregation. The corresponding statistical data is displayed in (B). Data are expressed as mean ± standard error of the mean (n=4).

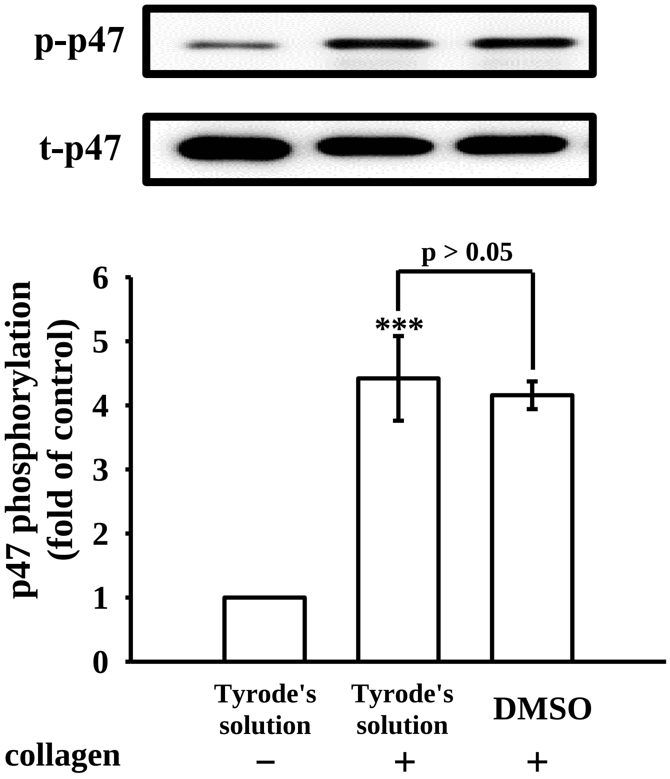

Figure S2. Effects of protein kinase C (PKC) activation in platelets. Washed platelets were preincubated with Tyrode’s solution or 0.1% DMSO and subsequently treated with collagen (1 μg/mL) to induce PKC activation (p-p47). Data are presented as the mean ± standard error of the mean (n = 4). ***p <0.001 compared with the resting platelets (Tyrode’s solution).
